# Sex differences in asthma: omics evidence and future directions

**DOI:** 10.3389/fgene.2025.1560276

**Published:** 2025-03-05

**Authors:** Bichen Peng, Weiyi Ye, Shuai Liu, Yue Jiang, Ziang Meng, Miao Guo, Lili Zhi, Xiao Chang, Lei Shao

**Affiliations:** ^1^ College of Medical Information and Artificial Intelligence, Shandong First Medical University and Shandong Academy of Medical Sciences, Jinan, Shandong, China; ^2^ Agricultural Products Quality and Safety Center of Ji’nan, Jinan, Shandong, China; ^3^ School of Life Sciences, Shandong First Medical University and Shandong Academy of Medical Sciences, Jinan, Shandong, China; ^4^ Department of Allergy, Shandong Institute of Respiratory Diseases, The First Affiliated Hospital of Shandong First Medical University, Jinan, Shandong, China; ^5^ The First Affiliated Hospital of Shandong First Medical University, Jinan, Shandong, China; ^6^ Department of infectious Disease, Central Hospital Affiliated to Shandong First Medical University, Jinan, Shandong, China

**Keywords:** asthma, sex differences, multi-omics, genomics, transcriptomics, epigenomics, proteomics, metabolomics

## Abstract

Asthma is a common and complex heterogeneous disease, with prevalence and severity varying across different age groups and sexes. Over the past few decades, with the development of high-throughput technologies, various “omics” analyses have emerged and been applied to asthma research, providing us with significant opportunities to study the genetic mechanisms underlying asthma. However, despite these advancements, the differences and specificities in the genetic mechanisms of asthma between sexes remain to be fully explored. Moreover, clinical guidelines have yet to incorporate or recommend sex-specific asthma management based on high-quality omics evidence. In this article, we review recent omics-level findings on sex differ-ences in asthma and discuss how to better integrate these multidimensional findings to generate further insights and advance the precision and effectiveness of asthma treatment.

## 1 Introduction

Asthma is a common chronic inflammatory airway disease with heterogeneity, and its etiology is complex, involving the interaction of environmental and genetic factors (Martinez, 2007; Tyler and Bunyavanich, 2019). The distribution of asthma patients varies not only between countries and regions but also shows significant differences in sex and age (Chowdhury et al., 2021; Osman, 2003; Zein and Erzurum, 2015; Tan et al., 2015). The prevalence of asthma is higher in boys than in girls before puberty, but this trend reverses in adulthood, with an increase in both prevalence and severity of asthma in women. Overall, asthma is more prevalent in women and is more likely to develop into severe asthma (Chowdhury et al., 2021; Zein et al., 2019; Wang et al., 2020). In addition to epidemiological and clinical studies indicating that the prevalence and severity of asthma are influenced by sex, findings from animal models also support the notion of sex differences in asthma. Specifically, during the development and progression of asthma, the pathophysiological responses of the airways suggest significant sex differences. Male mice exhibit greater airway hyperresponsiveness compared to female mice (McKenzie et al., 2010), which display higher levels of airway inflammation and lung remodeling (Blacquiere et al., 2010; Antunes et al., 1985). Furthermore, the European Network for Understanding Mechanisms of Severe Asthma (ENFUMOSA) and the Severe Asthma Research Program (SARP) found that women with severe asthma tend to have a higher BMI than those with non-severe asthma, a relationship not observed in men (Jarjour et al., 2012; Moore et al., 2010; Author Anonymous, 2003).

**FIGURE 1:**
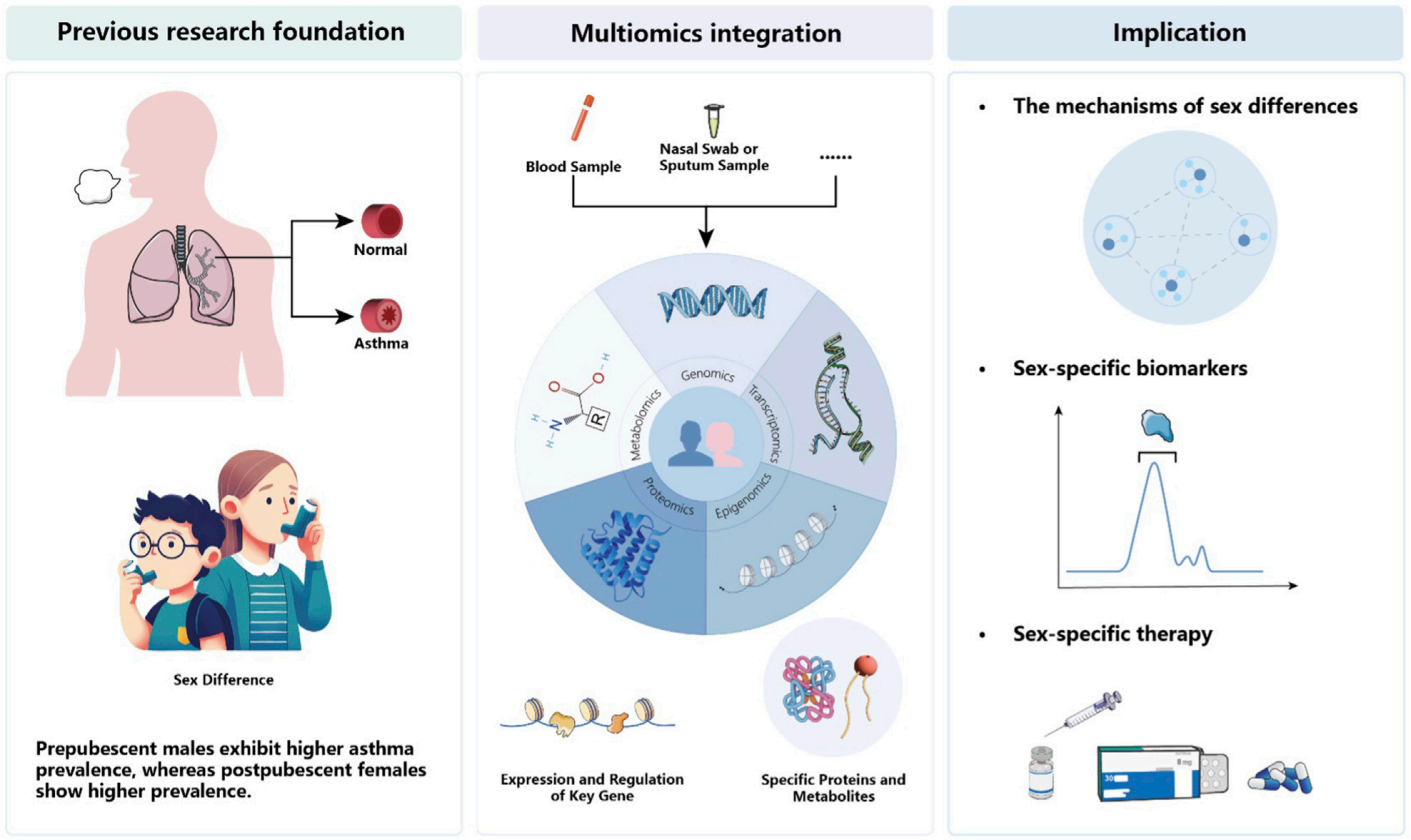
Multi-omics integration of sex-specific asthma studies.

While it is widely acknowledged that genetic susceptibility and exposure differences contribute to the heterogeneity of asthma manifestations (Tang et al., 2020), the underlying interactions between these determinants remain unclear, and the reasons for sex differences in asthma are not fully understood. Previous studies suggest that sex differences in asthma may be related to immune responses, sex hormones, and sex-specific responses to environmental exposures (Antunes et al., 1985; Becklake and Kauffmann, 1999; Almqvist et al., 2008; Vink et al., 2010; Melgert et al., 2007). These findings substantiate that sex plays a crucial role throughout the life cycle in the development and progression of asthma. However, in the era of precision medicine, merely recognizing the existence of sex differences in asthma is insufficient. Modern medicine requires causal inference rather than merely identifying associations (Richens et al., 2020). Moreover, there is a significant lack of clinically meaningful biomarkers to assist in the personalized diagnosis and treatment of asthma. To bridge this gap, multi-omics analysis methods, and mathematical models based on multi-omics data are needed (Figure 1).

In recent years, with the rapid development of omics technologies, our understanding of the molecular mechanisms of asthma has deepened. Particularly in sex difference studies, the application of omics technologies has revealed some key findings. To date, genomics and transcriptomics have identified over 60 genetic variants associated with sex differences in asthma and more than 300 differentially expressed genes with sex specificity. While recognizing the clinical utility of these findings remains challenging, especially in integrating multi-dimensional omics data and constructing a multi-omics model that links clinically meaningful elements at different levels, these challenges drive researchers to explore and seek new methods better suited to solving such unknown problems.

This review aims to systematically integrate and examine current research findings in the field of asthma, focusing on genomics, transcriptomics, epigenomics, proteomics, and metabolomics studies related to sex differences. By summarizing the results of multi-omics analyses, we explore the potential molecular mechanisms underlying sex differences in asthma and the current status of multi-omics research in this field. We hope to provide new theoretical perspectives for understanding sex differences in asthma and to promote sex-specific multi-omics research, ultimately advancing the development and implementation of predictive, preventive, and personalized medicine strategies tailored to sex differences in asthma.

## 2 Genomics

Genomic technologies involve systematic research on the genome of an organism, including the identification, location, and functional analysis of genes, and their roles in the development and diseases of the organism. This technology enables large-scale analysis of the entire genome through the application of high-throughput sequencing and bioinformatics tools (Hawkins et al., 2010). In recent years, genomic technologies have revealed numerous genetic variations associated with sex differences in asthma. These sex-specific genetic markers are not limited to the sex chromosomes (Cheong et al., 2005; Marques et al., 2017), but are widely distributed on autosomes (Table 1). Although the heterogeneity and complexity of asthma make it difficult to widely replicate the findings in existing literature, and some results are even contradictory, these studies still provide multi-faceted insights that significantly enrich our understanding of sex differences in asthma. For example, chromosome region 17q12-q21 is one of the most frequently confirmed regions in genome-wide association studies (GWAS) for asthma (Abdel-Aziz et al., 2020). A study based on the Canadian population reported a negative association between two SNPs (rs9303277, rs4795405) in this region and asthma, which was observed only in male subjects (Naumova et al., 2013). Specifically, rs9303277 may exert its effect by regulating the expression of genes associated with immune and inflammatory responses, such as the TLR2 gene. Mediation analysis suggests that 34%–36% of its effect on multi-trigger asthma is mediated through changes in TLR2 expression (Laubhahn et al., 2022). rs4795405 appears to influence the expression of the major asthma candidate gene ORMDL3, thereby regulating sphingolipid metabolism and endoplasmic reticulum stress responses, which in turn modulate Th2-mediated inflammation and affect asthma severity (Schedel et al., 2015). Another smaller-scale study on asthma in the Czech population reported that a haplotype consisting of four SNPs (rs17608925, rs12603332, rs8076131, and rs3169572) in this region was associated with allergic asthma in adult males but not in females (Hrdlickova and Holla, 2011). And these SNPs may also influence asthma onset by regulating the expression of ORMDL3 (Galanter et al., 2008; Ono et al., 2020). However, some asthma studies involving multiple ancestral groups did not find sex-specific genetic markers in this region (Halapi et al., 2010; Espuela-Ortiz et al., 2021). Clearly, such contradictions do not directly negate the sex differences in asthma.

**TABLE 1:** Male-specific loci identified based on UKBB/GBMI.

Chr	BP	SNP	Gene symbol	P_Male	P_Female	Database
1	8,656,434	rs1809332	RERE	1.64E-08	0.343	GBMI
5	132,009,599	rs17772853	IL4	3.20E-08	0.05825	GBMI
6	253,272	rs12193595	DUSP22	4.29E-08	0.07616	GBMI
7	22,763,845	rs2002792	IL6	1.97E-09	0.115	GBMI
8	128,955,726	rs551867916	MYC	6.25E-09	0.4971	GBMI
16	11,275,128	rs45487900	RMI2	4.79E-08	0.3171	GBMI
18	67,845,195	rs9963873	RTTN	2.55E-08	0.6636	GBMI

Studies have also attempted to use Genetic Risk Score (GRS) and Polygenic Risk Score (PRS) to assess the genetic risk of asthma in different sexes, consistently observing that the genetic risk scores are significantly more associated with asthma in males than in females (Sordillo et al., 2021; Han et al., 2020). After further controlling for age variables, the use of PRS to separately assess the genetic risks of childhood-onset asthma (COA) and adult-onset asthma (AOA) revealed that although males had a higher overall risk for COA, the correlation between PRS and asthma risk was higher in females. In AOA, the results were completely opposite (Espuela-Ortiz et al., 2021). These findings are consistent with epidemiological changes in asthma prevalence.

Early genetic studies on sex differences in asthma mostly focused on single cohort samples, observing the sex-specific impact of specific genetic variants on asthma and its related phenotypes (Ulbrecht et al., 2000; Santillan et al., 2003; Guerra et al., 2005; Szczeklik et al., 2004). For example, several studies have reported that the Beta-2 adrenergic receptor (β2 AR) is associated with asthma onset and has sex specificity (Ulbrecht et al., 2000; Santillan et al., 2003; Guerra et al., 2005). M. Ulbrecht et al. observed a protective effect of the Gly16-Gln27-Thr164 haplotype on bronchial hyperresponsiveness only in females in an East German population (Ulbrecht et al., 2000). Alfredo A. Santillan et al. found that Mexican adult males carrying the “Gly16 without Glu27 allele” had a significantly increased risk of asthma diagnosis, while a strong and independent negative association between the “Gly16-Glu27 allele” and asthma was observed only in female subjects (Santillan et al., 2003). Similarly, in adolescent populations, boys homozygous for Gly16 were twice as likely to have persistent wheezing compared to those with other β2 AR-16 genotypes (RR = 2.17; 95% CI = 1.41 to 3.36; p = 0.0003). In contrast, there was no association between β2 AR-16 genotype and persistent wheezing in girls (Guerra et al., 2005).

As our understanding of racial differences in asthma deepens (Mannino et al., 2002; Author Anonymous, 1997; Lester et al., 2001), and with advancements in statistical analysis techniques, researchers have gradually attempted to conduct genetic studies on sex differences in asthma across multiple ethnic groups. These studies often include sex stratification and interaction analyses, incorporating indicators such as lung function parameters, serum IgE levels, and eosinophil counts to further explore sex differences in asthma.

A genomic study based on cohorts including African Americans, European Americans, and Hispanics found that rs2702945 in the intron region of the DEFB1 gene showed low transmission of the A allele only in European American female asthma cases (P = 0.007). This may indicate a protective effect of this variant in reducing the risk of asthma in European American females. Notably, rs2741136 in the 5′region also showed low transmission of the C allele in European American female subjects, with a transmission ratio of 6:7 (P = 0.054). Although this P value did not reach the commonly used statistical significance threshold (P ≤ 0.05), the result was very close, suggesting that with an increased sample size, this SNP might reach significant sex-specific transmission bias (Levy et al., 2005). Another study involving European American, African American, African Caribbean, and Latino populations reported six sex-specific asthma risk loci, all of which were ancestry-specific. For example, rs2549003 near the IRF1 gene at 5q31.1 showed a sex-specific association only in European American males. The rs17642749 at 10q26.11 was male-specific in African American/African Caribbean samples, while rs1012307, rs9895098, rs4673659, and rs2675724 at 2q23.3 were specific risk loci for Latino females (Myers et al., 2014). Among these, rs2549003 and rs9895098 have been shown to be associated with asthma in the GABRIEL Consortium meta-analysis of asthma in European subjects (Moffatt et al., 2010). However, an analysis based on German and Austrian, Turkish, Dutch, and Russian populations failed to replicate the sex-SNP interaction results for rs2549003 in European Americans. In their dataset, no sex-SNP interaction trend or asthma association was observed in male subjects for rs2548997, which is described as being in complete linkage disequilibrium with rs2549003 (Schieck et al., 2016). It is important to consider that studies focusing only on sex stratification might overlook genetic loci with sex interaction effects (Espuela-Ortiz et al., 2021; Mersha et al., 2015). Future asthma genomics research may need to pay more attention to sex interactions. Moreover, the above results are ethnicity-specific, highlighting the importance of examining genetic markers in different genetic backgrounds. To enhance the generalizability of the research and the reliability of the findings, future studies should further increase sample sizes and adopt multi-center study designs that encompass more ethnicities and geographic regions, in order to validate these findings and explore their applicability in asthma patients worldwide.

A study that conducted quantitative trait analyses on intermediate phenotypes related to asthma, such as post-bronchodilator FEV1, bronchodilator (BD) response, post-BD FEV1/FVC ratio, airway hyperresponsiveness to methacholine, total serum IgE levels, and total serum eosinophil counts, reported no significant associations between VDR polymorphisms and phenotypes in mixed-sex analyses. However, sex-stratified analyses showed that rs2239179 and rs1540339 were associated with total serum IgE levels and total serum eosinophil counts in girls, rs2239185 was associated with total serum IgE levels and FEV1/FVC ratio in girls, rs731236 (TaqI) was associated with total serum IgE levels in girls, and rs2228570 (FokI) was associated with total serum eosinophil counts and FEV1/FVC ratio in boys (Raby et al., 2004). These sex-specific SNPs were associated with multiple intermediate phenotypes, indirectly demonstrating the complexity of asthma’s pathological mechanisms. Another study exploring the relationship between sex hormone receptor gene polymorphisms and asthma found that SNPs in the estro-gen receptor alpha gene were significantly associated with susceptibility to airway hyperresponsiveness (AHR) and faster lung function decline in females. Notably, IVS1-397 C/T polymorphism (with the T allele being the risk allele) showed the strongest association, along with exon1+30 T/C and IVS1-351 G/A polymorphisms, only in female subjects (Dijkstra et al., 2006).

Lastly, we analyzed asthma-related data from the UK Biobank (UKBB) and the Global Biobank Meta-analysis Initiative (GBMI). First, we performed CLUMP analysis on the asthma-associated loci data and calculated the linkage disequilibrium between male and female loci, excluding loci with *r* ^2^ greater than 0.1. Ultimately, we identified multiple sex-specific loci (significance threshold of 5E-8, P-value greater than 0.05 for the non-significant sex), as shown in Tables 1, 2. In addition to the genes TNFAIP3, ZFP36L1, and IL4/IL6, which have been identified in multiple studies as related to asthma, we also found some interesting results. For example, the gene corresponding to rs12262856, SORCS1, is related to obesity and metabolism. As an intracellular transport receptor, it regulates brain-derived neurotrophic factor (BDNF) signaling and, together with SORCS3, controls the production of the appetite peptide AgRP in hypothalamic arcuate nucleus neurons, thereby affecting food intake and energy balance. The absence of SORCS1 can lead to increased food intake, reduced physical activity, decreased fat utilization, and increased fat accumulation (Subkhangulova et al., 2018). The co-occurrence trend of obesity and asthma and socioeconomic factors are considered potential mechanisms linking the two. Further studies have indicated that obesity may increase asthma risk through various mechanisms, including increased systemic and local inflammation, altered adipokine levels, and changes in dietary structure (Black and Sharpe, 1997).

**TABLE 2:** Female-specific loci identified based on UKBB/GBMI.

Chr	BP	SNP	Gene symbol	P_Female	P_Male	Database
2	24,844,422	rs55751342	NCOA1	3.24E-09	0.3199	GBMI
2	203,398,944	rs1199498	FAM117B	4.07E-08	0.6433	GBMI
3	49,902,160	rs2883059	CAMKV	2.27E-13	0.1716	GBMI
3	50,153,356	rs1138536	RBM5	3.14E-12	0.262	GBMI
3	109,360,913	rs564585335	DPPA4	2.28E-08	0.384,536	UKBB
6	90,898,649	rs113191185	BACH2	2.56E-08	0.08998	GBMI
6	138,135,621	rs569305282	TNFAIP3	1.14E-09	0.8008	GBMI
8	22,473,013	rs148396036	CCAR2	1.32E-08	0.393,592	UKBB
10	108,215,447	rs12262856	SORCS1	3.43E-08	0.8773	GBMI
11	28,627,504	rs10835363	METTL15	9.70E-09	0.05057	GBMI
11	61,090,585	rs56358658	TKFC	8.90E-09	0.881,777	UKBB
12	123,908,290	rs28376121	RILPL2	1.86E-09	0.05317	GBMI
14	69,177,415	rs61641915	ZFP36L1	3.53E-08	0.07749	GBMI
15	41,783,814	rs1757463	ITPKA	1.60E-08	0.5184	GBMI
15	49,948,824	rs11852336	DTWD1	1.62E-08	0.07104	GBMI
17	66,008,044	rs62086897	C17orf58	2.68E-08	0.3562	GBMI
17	73,499,981	rs143966283	CASKIN2	2.41E-10	0.56056	UKBB
18	77,561,978	rs8091998	KCNG2	1.39E-10	0.9881	GBMI

**FIGURE 2:**
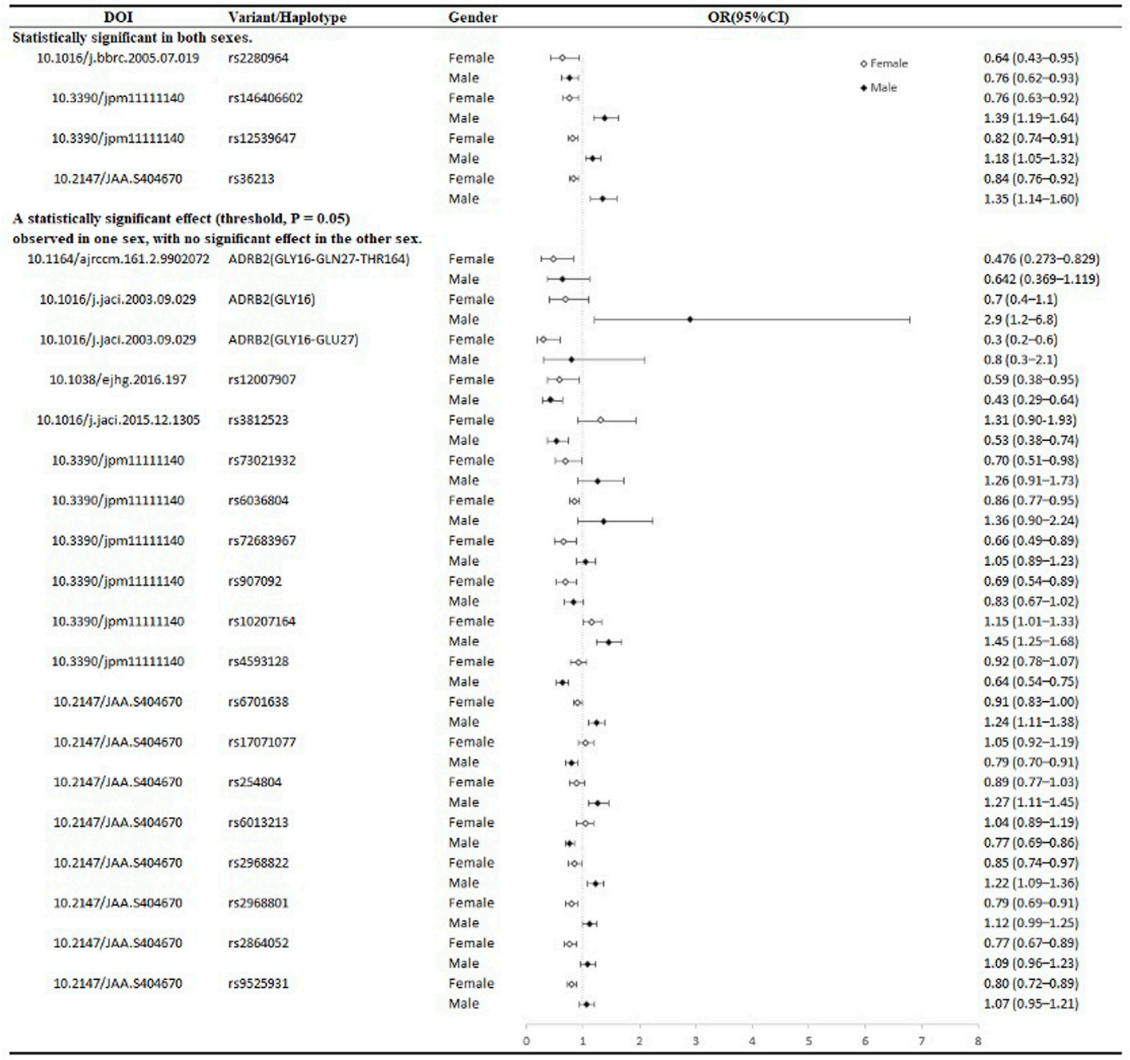
Forest plot of sex differences in asthma-related variants from literature.

To date, numerous studies have reported sex differences in the association between single nucleotide polymorphisms (SNPs) on different chromosomes and intermediate phenotypes reflecting asthma or its severity, such as IgE levels, FEV1/FVC ratio, and serum eosinophil counts. These sex differences are mainly reflected in two aspects: first, in some cases, although both sexes show similar associations, the significance is reached only in a single sex group; second, some SNPs exhibit opposite effects on intermediate phenotypes in different sexes (Lee et al., 2023; Seibold et al., 2008; Aschard et al., 2009; Hunninghake et al., 2008), the forest plot in Figure 2 provides a detailed depiction of these sex differences as reported in previous studies. Clearly, focusing solely on such associations is not sufficient. For example, IgE is widely recognized as an important biomarker for allergic diseases, including asthma (Hammad and Lambrecht, 2021). Although CTLA-4 (+49 A/G) is associated with IgE levels in Asian females (Yang et al., 2004; Chang et al., 2004; Roshanizadeh et al., 2021), no significant association has been shown between this polymorphism and asthma severity (Roshanizadeh et al., 2021; Lee et al., 2002). These results reveal the complexity and variability of asthma and its underlying mechanisms, highlighting the importance of considering multiple variables and broader genetic backgrounds, even integrating multi-omics analyses in asthma research, Table 3 summarizes the sample characteristics and variant information from multiple studies, further supporting the importance of sex-specific variations in asthma and related diseases in previous studies.

**TABLE 3:** Overview of sample characteristics and variant information of sex-specific variations related to asthma from literature.

Total sample,NO.	Females,%	Variant/Haplotype	Chr	Disease/Trait	Reference (DOI)
165	51	ADRB2 (Gly16/Glu27/THR16)	5	Bronchial hyperresponsiveness	10.1164/ajrccm.161.2.9902072
907	66	ADRB2 (Gly16/Gly16-Glu27)	5	Asthma	10.1016/j.jaci.2003.09.029
271	38.7	ADRB2 (Gly16)	5	Asthma/Wheezy	10.1378/chest.128.2.609
857	60	COX-2 (G-765C)	1	Asthma	10.1016/j.jaci.2004.05.030
1,333	50	CTLA-4 (+49 A/G)	2	Allergic disorders	10.1111/j.1365-2222.2004.01776.x
644	47	CTLA-4 (+49 A/G)	2	Allergic disorders	10.1111/j.1399-3038.2004.00161.x
637	37.4	rs2239179 rs1540339 rs2239185 rs731236 rs2228570	12	Asthma	10.1164/rccm.200,404OC-447OC
129	41	ESR1 (IVS1-397 C/T、exon1+30 T/C、IVS1-351 G/A)	12	Asthma/Bronchial hyperresponsiveness	10.1016/j.jaci.2005.11.023
516	38.2	rs2702945 rs2741136	8	Asthma	10.1016/j.jaci.2004.11.013
1,390	59.8	rs2280964	X	Asthma	10.1016/j.bbrc.2005.07.019
1,307	46.1	rs12007907	X	Asthma	10.1038/ejhg.2016.197
920	37.9	rs2289276	5	Asthma	10.1164/rccm.200,711OC-1697OC
12,071	51.5	rs1837253 rs2289276	5	Asthma	10.1111/j.1398-9995.2010.02415.x
727	46.9	rs2069882 rs2069885 D11S925 D5S2115	5	Asthma	10.1038/gene.2009.46
509	60.9	KCNMB1 c.818C>T	5	Asthma	10.1093/hmg/ddn168
1,214	54	rs9303277 rs4795405	17	Asthma	10.1007/s00439-013-1298-z
9049	51.9	rs2549003 rs17642749 rs1012307 rs4673659 rs2675724 rs9895098	2,5,6,10,17	Asthma	10.1093/hmg/ddu222
4229	37.4	rs3812523	9	Asthma	10.1016/j.jaci.2015.12.1305
4843	52.1	rs10971545 rs7848134 rs59070393	9.22	Asthma	10.1093/aje/kwy278
699	47.8	rs9784675 rs2243274 rs2303064 rs12201458 rs9325071 rs7445392 rs2303063 rs2069884 rs1051052 rs510432 rs6892205 rs1322178 rs4701999 rs2997049 rs2227562 rs11102221 rs6498011 rs3804329 rs4065 rs6968671 rs4263489 rs1057258 rs7525758 rs290986 rs3740689 rs895314 rs723680 rs17421942 rs3024676 rs2275913 rs12006123 rs1423007 rs2289277 rs290988 rs17407962 rs3822744 rs680925 rs2248849	1,2,5,6,7,9,10,11,14,16,20,23	Asthma	10.1016/j.ygeno.2015.03.003
6,021	51.1	rs146406602 rs73021932 rs6036804 rs12539647 rs72683967 rs4400476 rs907092 rs10207164 rs4593128	2,4,6,7,8,9,17,20	Asthma	10.3390/jpm11111140
400	66	rs231775	2	Asthma	10.1080/15,257,770.2021.1964525
26,622	49.9	rs6701638 rs17071,077 rs254804 rs6013213 rs2968822 rs2968801 rs2864052 rs9525931 rs36213	1,2,3,6,13,19,20	Asthma	10.2147/JAA.S404670
1,173	39.1	rs1255383	10	Asthma/Atopy	10.1183/13,993,003.00479-2022

## 3 Transcriptomics

Building upon genomics, transcriptomics provides us with in-depth insights into how genes are activated or suppressed across different sexes, physiological states, and stages of disease by analyzing RNA molecules. This shift from static genetic sequences to dynamic gene expression patterns not only further enriches our understanding of the pathogenesis of asthma but also offers new strategies for identifying sex-specific therapeutic targets (Wang et al., 2009). As demonstrated in the genomics section, sex differences play a key role in the development of asthma, and transcriptomic research will further explore how sex influences the gene expression patterns of asthma patients. This helps researchers uncover the specific roles of genes with significant expression differences between sexes in the pathogenesis of asthma.

A study combining genomics and transcriptomics revealed that sex specificity in asthma can be observed at both levels of analysis. Specifically, the study identified 47 sex-specific asthma-related SNPs and 29 sex-specific asthma-related probes. Addition-ally, the study found that more than half (55%) of the genetic variations associated with asthma identified in sex-specific analyses were not detected in sex-combined analyses. Similarly, at the transcriptomic level, nearly one-third (31%) of the probes were only detected in sex-specific analyses and not identified in broader sex-combined analyses (Mersha et al., 2015). Furthermore, a transcriptomic study based on 711 males and 689 females across five different tissues and cell types (epithelial cells, blood, induced sputum, T cells, and lymphoblastoid cells) identified 332 significantly differentially expressed genes (DEGs) associated with asthma in males and 87 DEGs in females, with only 19 genes overlapping between the sexes. By correlating the identified DEGs with asthma-related SNPs extracted from the GWAS catalog, the study integrated genomic and transcriptomic information, ultimately focusing on four male-specific genes (FBXL7, ITPR3, and RAD51B from epithelial tissue and ALOX15 from blood) and one female-specific gene (HLA-DQA1 from epithelial tissue) (Gautam et al., 2019). Notably, FBXL7 is often hypomethylated (upregulated) in atopic patients (Forno et al., 2019). Its expression is also associated with a reduced response to inhaled corticosteroids in asthma patients (Park et al., 2014). Additionally, ALOX15-induced lipid peroxidation has been shown to increase the susceptibility of asthma epithelial cells to ferroptosis (Zhang et al., 2024). By combining data from genomics and transcriptomics, studies can more accurately identify key disease-related genes, making the results more reliable and clinically meaningful.

As we know, total serum immunoglobulin E (IgE) levels are widely recognized as an intermediate phenotype associated with both asthma onset and severity (Burrows et al., 1989; Sears et al., 1991). Sex is also a significant determinant of IgE levels, with males generally having higher total IgE levels than females (Guilbert et al., 2004; Raby et al., 2007). A study linking CD4^+^ T cell transcript abundance with human IgE levels found that in sex-stratified analyses, IL17RB transcript abundance was correlated with IgE levels in males (r2 = 0.19) but not in females (r2 = 0.03). Furthermore, sex-stratified IPA (Ingenuity Pathway Analysis) of canonical pathways revealed different enrichment patterns between males and females. For instance, 13 pathways showed nominal enrichment evidence in males, while six pathways, including the interferon signaling pathway, were enriched in females. Notably, no significant canonical pathways overlapped between males and females. These sex differences suggest that asthma and other allergic diseases may have different molecular mechanisms in different sexes (Hunninghake et al., 2011).

Given that the susceptibility to asthma changes with sex differences before and after puberty (Vink et al., 2010; Teague et al., 2018), as well as the acute exacerbations of asthma observed during the menstrual cycle (Eliasson et al., 1986; Oertelt-Prigione, 2012), coupled with the gradually revealed sexual dimorphism of the immune system (Klein and Flanagan, 2016), an increasing number of transcriptomic studies have focused on the role of pubertal development and sex hormones. For example, a study exploring the overall correlation of gene expression changes related to both puberty and asthma found sex-specific patterns. In males, gene expression changes related to puberty (and age) were negatively correlated with changes associated with asthma symptoms and positively correlated with changes in lung function. Conversely, in females, gene expression changes related to puberty (and age) were positively correlated with changes associated with asthma symptoms and negatively correlated with changes in lung function. Moreover, the study identified 56 genes with differential expression in females before and after menarche among the 1,621 genes associated with asthma risk, with four genes (IKZF4, GFI1, RP11-111M22.2, IL21R) showing changes in expression during female pubertal progression (Resztak et al., 2023). Another study suggests that early menarche (<11 years) further increases the risk of asthma in females (Fuseini and Newcomb, 2017). A transcriptomic study based on mouse models suggested that androgen signaling might reduce asthma risk in male mice by inducing DUSP-2 to suppress Th2 cell cytokine production (Ejima et al., 2022). Although the specific mechanisms by which sex hormones regulate the development and severity of asthma remain unclear, existing transcriptomic evidence suggests that sex hormones do play a significant role in this process.

Furthermore, as a disease influenced by both environmental and genetic factors (Miller et al., 2021; Bonnelykke and Ober, 2016), asthma shows sex differences at the transcriptomic level regarding environmental exposures. A study found sex-specific associations between intrauterine smoke (IUS) exposure and miRNA expression. By including sex and IUS interactions in the regression model, the researchers identified 95 significant IUS-miRNA associations in male samples, compared to only four in female samples. Additionally, miR-101-3p expression significantly decreased with IUS exposure in all samples and male samples, but not in female fetal lung samples. Further research indicated that decreased miR-101-3p expression was associated with increased allergen response to house dust mites and bronchodilator response in children with asthma, findings observed in all samples and male subjects but not in female subjects (Rosenberg et al., 2023). Another study examining the impact of *in utero* exposure to e-cigarette aerosols on lung development and asthma susceptibility in mouse offspring also showed significant sex differences. Among male mice, 88 genes were significantly regulated (62 upregulated and 26 downregulated), while among female mice, 65 genes were significantly regulated (17 upregulated and 48 downregulated). *In utero* exposure to e-cigarette aerosols exacerbated house dust mite (HDM)-induced asthma responses in male offspring at 7 weeks of age. IPA analysis indicated that affected gene pathways in male mice included CD28 signaling in helper T cells, NFAT regulation of immune response, and phospholipase C signaling, while genes affected in female mice were mainly related to NRF2-mediated oxidative stress response. This suggests that *in utero* exposure to e-cigarette aerosols may alter lung development and immune response states differently in male and female mice (Noel et al., 2023). Interestingly, in addition to the sex differences in offspring asthma risk due to intrauterine smoke or e-cigarette aerosol exposure, placental gene expression in asthma pregnancies also shows sex-specific changes (Scott et al., 2009). A transcriptomic study indicated significant differences in the quantity and type of gene expression changes in male and female placentas when the mother had asthma. In female placentas, gene expression changes related to growth and development were particularly prominent (Osei-Kumah et al., 2011). This partly explains the phenomenon where untreated maternal asthma during pregnancy is associated with low birth weight only in female fetuses, while male fetal birth sizes remain normal (Murphy et al., 2003), Table 4 summarizes the characteristics of human datasets used in sex-specific transcriptomic research on asthma, providing crucial support for a deeper understanding of sex-specific differences in asthma.

**TABLE 4:** Characterization of human datasets utilized in transcriptomics research.

GEO (dbGaP) accession	Sample size (male/female)	Sample type	Reference (DOI)
GSE8052	404 (221/183)	Peripheral blood Lymphocytes	10.1016/j.ygeno.2015.03.003
GSE64913	70 (41/29)	Central/Peripheral airway epithelium	10.1093/hmg/ddz074
GSE43696	58 (19/39)	Fresh bronchial epithelial cells	10.1093/hmg/ddz074
GSE65204	69 (34/35)	Nasal epithelial cells	10.1093/hmg/ddz074
GSE89809	43 (27/16)	Endobronchial epithelial brushings/CD3+ T-cells	10.1093/hmg/ddz074
GSE69683	333 (138/195)	Blood	10.1093/hmg/ddz074
GSE27011	35 (25/10)	White blood cells	10.1093/hmg/ddz074
GSE35571	124 (76/48)	Peripheral blood sample	10.1093/hmg/ddz074
GSE40732	194 (97/97)	Peripheral blood mononuclear cells	10.1093/hmg/ddz074
GSE76262	70 (33/37)	Induced sputum	10.1093/hmg/ddz074
GSE8052	404 (221/183)	Peripheral blood lymphocytes	10.1093/hmg/ddz074
GSE22324	200 (121/79)	Blood	10.1186/1471-2466-11-17
phs002182 v2.p1	251 (148/103)	Blood	10.1038/s41467-022-35742-z
GSE200153	298 (166/132)	Fetal lung tissue	10.3390/ijms24097727

However, the limitation of transcriptomics lies in its provision of gene expression level data alone, without elucidating the genetic regulatory mechanisms underlying these changes or the impact of environmental factors. Therefore, to gain a comprehensive understanding of the complex pathophysiological mechanisms of asthma, and to achieve predictive, preventive, and personalized medicine, incorporating epigenomic research is essential.

## 4 Epigenomics

Epigenomics focuses on studying heritable changes in gene expression that do not involve alterations to the DNA sequence. Instead, these changes are achieved through mechanisms such as DNA methylation, histone modification, and chromatin remodeling. These mechanisms play a crucial role in cellular differentiation, development, and the onset of diseases, providing in-depth insights into the complexity of gene function and its role in a variety of diseases (Wang and Chang, 2018).

For instance, a study examined the relationship between DNA methylation that varies by sex and age in the chromosomal region 17q12-q21 and its connection to childhood asthma. This research identified significant genetic links in the 17q12-q21 region specifically in male asthma patients, contrasts that were less evident in female patients. Through meticulous analysis of the methylation profiles of the promoters of five genes within the 17q12-q21 region, researchers identified a single regulatory region within the ZPBP2 gene that showed statistically significant methylation differences between males and females. Additionally, the study found that DNA methylation levels varied with age, particularly in adult males, where methylation levels were higher than in male children (Naumova et al., 2013). This finding suggests that the decrease in asthma prevalence in males after puberty may be associated with an increase in DNA methylation levels of the ZPBP2 gene in adulthood, which could potentially influence genetic susceptibility to asthma by modulating gene expression.

Furthermore, another study has revealed that the sex reversal phenomenon in asthma onset is closely related to changes in DNA methylation (DNAm) at asthma-related cytosine-phosphate-guanine (CpG) sites from before to after puberty, with opposite trends observed in males and females. By conducting an in-depth analysis of DNAm in adolescents from the Isle of Wight Birth Cohort (IOWBC), researchers identified 535 CpG sites that may be associated with asthma. Notably, significant interactions between sex and DNAm changes were observed at 13 CpG sites, a finding that was validated in the Avon Longitudinal Study of Parents and Children (ALSPAC) cohort (Patel et al., 2021).

Although epigenomics has provided some explanations for the sex differences in asthma incidence and the reversal of these differences before and after puberty, the pathogenesis of asthma is highly complex and cannot be fully understood at the epigenetic level alone. To comprehensively elucidate the multi-level molecular mechanisms underlying sex differences in asthma and to further advance the prediction, prevention, and personalized medicine for asthma, future research should adopt a multi-omics approach.

## 5 Proteomics and metabolomics

The discovery of new candidate protein biomarkers has become a major research focus in the fields of clinical chemistry, analytical chemistry, and biomedical science (Alexovic et al., 2020). Proteins, being direct executors of biological functions, reflect the transient state of tissues/cells during the study. Understanding the biological regulatory processes at the protein level is crucial for comprehending the molecular biological basis of diseases and for more precise typing. Proteomics is the study of the expression, structure, function, interaction, and modification of a set of proteins present under specific times or conditions (Li et al., 2017).

Proteomic analysis plays a significant role in asthma research, often identifying potential protein biomarkers related to different asthma phenotypes through the analysis of biological samples such as serum, plasma, sputum, and bronchoalveolar lavage fluid (Kere et al., 2023; Asamoah et al., 2024; Hastie et al., 2024). However, to date, no proteomic studies have systematically explored the sex differences in asthma patients. A multi-omics study utilizing sputum transcriptomics and proteomics aimed to explore population differences after clustering mid to severe asthma patients based on a series of clinical physiological parameters. The study successfully identified four stable and replicable asthma patient groups, showing significant differences in sputum proteomics and transcriptomics.

Notably, one group in the study mainly included high BMI female patients who had not effectively controlled their asthma. Although their lung function remained within the normal range, they frequently experienced asthma exacerbations. Moreover, compared to the group of severe asthma patients with airway obstruction and oral corticosteroid (OCS) use but no history of smoking, the Cathepsin G levels were significantly higher in this high BMI female group (Lefaudeux et al., 2017). While Cathepsin G levels were also high in the well-controlled asthma group, the elevated Cathepsin G levels in patients experiencing frequent exacerbations and uncontrolled symptoms may reflect a biological mechanism in this group for maintaining or enhancing inflammatory activity, possibly related to overactive or abnormal immune activation during asthma exacerbation. Furthermore, another study using an unsupervised clustering method for phenotyping severe asthma patients identified five groups, one of which was a hard-to-manage late-onset group, mainly consisting of elderly obese women frequently needing oral corticosteroid treatment (Moore et al., 2010). These findings suggest potential sex-specific differences in protein expression among asthma patients, highlighting the possible influence of sex factors in the pathobiology of asthma. Indeed, the scarcity of proteomic studies on sex-specificity in asthma patients may partly stem from the higher complexity and variability of the proteome compared to the genome and transcriptome (Smith, 2002). Although early proteomic studies explored differences in plasma proteomics between healthy pregnant women and those with asthma (Murphy et al., 2006), this suggests that women may constitute a sub-group with unique biological characteristics among asthma patients, and that sex and physiological status may influence the biomarkers and pathological processes of asthma.

Unlike the situation with proteomic studies, metabolomics has been more active in exploring sex-specificity in asthma. Indeed, metabolomics studies have begun to re-veal significant metabolic differences between male and female asthma patients, providing new biomarkers and potential therapeutic targets for understanding the sex differences in asthma. A lipidomic analysis from Peking University Third Hospital clearly indicated significant differences in the serum glycerophospholipid profiles be-tween adult asthma patients of different sexes, with lower levels of LPC, LPC(O), and LPS in female patients compared to male patients (Gai et al., 2018). Additionally, the team found in another study exploring differences in sphingolipid metabolism between male and female asthma patients that five types of ceramides (Cer16:0, Cer20:0, Cer22:0, Cer24:0, and Cer26:0) and one type of sphingomyelin (SM38:0) were significantly higher in male patients than in female patients, even among non-smokers (Song et al., 2021). This suggests that sex may be an important factor affecting sphingolipid metabolism. These differences could be related to different levels of sex hormones, as the study also found higher testosterone levels in male patients and higher estrogen levels in female patients. These sex hormones might influence lipid metabolism through different pathways. The study also discovered that Cer16:0 and Cer20:0 in male patients were significantly positively correlated with the inflammatory factor IL-5. In female patients, Cer20:0 was negatively correlated with the inflammatory factor IL-10, and Cer16:0 was negatively correlated with IL-5 (95). Known to promote the survival and activation of eosinophils, IL-5 is a key cytokine often associated with eosinophilic phenotypes and type 2 inflammation in asthma (Principe et al., 2021), while IL-10 is an immunosuppressive cytokine involved in regulating immune responses (Ren et al., 2021). Therefore, the positive correlation between Cer16:0 and Cer20:0 with IL-5 in male patients may imply that these ceramides play a role in promoting the expression or activity of IL-5, thereby enhancing eosinophilic responses and exacerbating allergic symptoms. However, these ceramides show a markedly different relationship with inflammatory factors in female patients. And generally, compared to males, females with asthma experience more severe symptoms and higher rates of ex-acerbation, hospitalization, and mortality (Mattiuzzi and Lippi, 2020; Senna et al., 2021; Boulet et al., 2022), which appears contradictory to the study results. But this may precisely reflect the sex differences in lipid metabolism in asthma inflammatory responses, and such relationships are complex.

Another metabolomics result suggested significant differences in the associations between asthma exacerbation frequency and metabolites in males and females. In males, the involved metabolites were primarily related to fatty acid and corticosteroid metabolism, while in females, they involved broader pathways including carbohydrate and amino acid metabolism, possibly reflecting fundamental differences in biochemical pathway activities in asthma between sexes (Kachroo et al., 2021). However, the small sample size in males in this study may have limited the ability to detect a broader range of metabolites. Additionally, a serum metabolomics study based on a guinea pig asthma model indicated significant differences in serum metabolites between male and female guinea pigs. Particularly in pathways like arachidonic acid metabolism, glycolysis, and glycerophospholipid metabolism, female guinea pigs showed higher sensitivity to asthma, and males and females showed opposite effects in regulating most metabolites (Liu et al., 2022). Another metabolomics study focused on the sex differences in plasma metabolites in a guinea pig asthma model, particularly noting significant differences in plasma concentrations of metabolites involved in energy acquisition pathways like lactate, glucose, alanine, and citrate between male and female guinea pigs (Barosova et al., 2023).

It is undeniable that most of the results from these metabolomics analyses focused on lipid metabolism. Lipid mediators are key drivers in asthma inflammatory responses, playing significant roles in T-cell recruitment and energy metabolism (Kelly et al., 2017). And compared to males, females with asthma are also more prone to metabolic disorders such as dyslipidemia and type 2 diabetes, although the reasons for this are not yet clear (Cazzola et al., 2011). Overall, these results further support the necessity of innovative development based on proteomics and metabolomics for asthma sex-specific biomarkers.

## 6 Discussion

“Sex” is increasingly emphasized in healthcare, and considering sex factors in the diagnosis, prevention, and treatment of diseases is a necessary and fundamental step towards precision medicine (Mauvais-Jarvis et al., 2020). Growing research shows that sex interacts with race/ethnicity and age in both healthy and diseased states (Assari et al., 2016; Assari et al., 2018; Kanchi et al., 2018), and sex differences exist across various types of diseases (Chowdhury et al., 2021; Klein and Morgan, 2020; Ye et al., 2020; Eastwood et al., 2023; Hester et al., 2019; Rexrode et al., 2022; Connelly et al., 2019). These findings will undoubtedly drive the development of sex-specific biomarkers and the generation of relevant evidence-based guidelines. Omics science is undoubtedly a powerful analytical tool. It can guide precise disease classification and biomarker development from the perspective of sex differences, and even evaluate treatment effects (Guo et al., 2023; Reel et al., 2022; Beheshti et al., 2022; DeMeo, 2021; Meoni et al., 2022).

Traditionally, asthma research has mostly been conducted through questionnaires or by analyzing laboratory parameters such as lung function tests and the cellular and mediators in various body fluids (blood, urine, sputum, bronchoalveolar lavage fluid). Therefore, before the advent of the “omics era,” asthma was mainly clinically diagnosed and classified based on clinical symptoms or relatively simple biomarkers. However, such clinical diagnoses are undoubtedly limited and subjective. For example, asthma can easily be confused with COPD and other respiratory diseases, and there is even a more complex disease known as asthma-COPD overlap (ACO) (Leung and Sin, 2017; Dasgupta et al., 2023). Omics technologies can help us delineate the molecular fingerprints of asthma (Pividori et al., 2019; Vieira Braga et al., 2019; Delgado-Dolset et al., 2022; Ghosh et al., 2020). Genomics studies DNA sequence variations to explore their associations and functional correlations with phenotypes. Many genomic studies have also clarified that asthma susceptibility differs between sexes.

Most genomic studies are observational (Bouchard, 2015), which means that the evidence they provide is only strong under certain conditions. Additionally, most genomic studies focus on reporting significant results, which may lead to the use of relatively loose cutoff p-values to find more significant SNPs. This practice needs to focus on the effect sizes of these SNPs (Vijverberg et al., 2018), although with larger sample sizes, these SNPs might also meet stringent cutoff p-values. Interestingly, aside from the 17q12-21 locus, other asthma GWAS results are often non-overlapping and non-replicable (Abdel-Aziz et al., 2020). Since the initial discovery of the 17q12 locus’s association with asthma (Moffatt et al., 2007), it has been repeatedly reported (Moffatt et al., 2010; Shrine et al., 2019; Wan et al., 2012; Karunas et al., 2011; Sleiman et al., 2010; Torgerson et al., 2011; Ferreira et al., 2011), and expanded to 17q12-21 (Moffatt et al., 2010). Even so, their exact roles in asthma have not been fully elucidated (Stein et al., 2018).

Genomic studies can usually only explain a small part of the heritability of complex human diseases (Eichler et al., 2010). Combining genomics with transcriptomics is expected to provide more information on how genetic variations regulate gene expression, revealing the complex molecular mechanisms of asthma. To date, many transcriptomic asthma studies covering various sample types have been conducted (Gautam et al., 2019; Vieira Braga et al., 2019; Baines et al., 2020; Zeng et al., 2023; Yan et al., 2024). These studies focus on differences in transcripts between asthma patients and healthy controls or among different asthma phenotypes, providing important insights into the role of gene expression differences in the development and progression of asthma. Some studies also combine genomics and transcriptomics to provide more information on how genetic variations regulate gene expression, which seems more helpful in revealing the complex molecular mechanisms of asthma (Pividori et al., 2019; Levin et al., 2019). Overall, different clinical phenotypes indeed have different transcriptomic characteristics (Levin et al., 2019; Hekking et al., 2018). Therefore, from the perspective of studying sex differences, we need to identify well-defined cohorts and control for appropriate confounding factors in the study design phase. This approach allows for more refined and objective asthma classification or biomarker development based on sex. Of course, transcriptomics can certainly be regarded as an intermediate layer in multi-omics research. Therefore, exploring asthma by combining genomics and transcriptomics is very necessary.

Compared to genomics and transcriptomics, proteomics and metabolomics are undoubtedly closer to disease phenotypes. Correspondingly, due to the complexity of asthma, proteomics may be more challenging to characterize. To date, no proteomics study has grouped subjects by sex in the experimental design phase but rather used sex as a covariate to adjust for potential baseline differences between groups (Kere et al., 2023; Gharib et al., 2011; Cao et al., 2017). However, from a cluster analysis in a multi-omics study, we can still see that high BMI women, as a special group, have a significantly different proteomic profile compared to other groups (Lefaudeux et al., 2017).

Metabolomics, relative to proteomics, is more downstream. Metabolomics focuses on analyzing low molecular weight biochemical compounds (<1,500 Da) related to metabolism (Ivanova et al., 2019). These compounds are closely related to the state of the organism, helping to maintain redox balance, and participating in oxidative stress, cell signaling, apoptosis, and inflammatory processes. As a result of gene expression and environmental factors, they can provide information on multi-factorial diseases such as asthma (Tao et al., 2019; Kelly et al., 2018). In terms of sex differences in lipid metabolism among asthma patients, metabolomics indeed provides direct evidence (Gai et al., 2018; Song et al., 2021). However, such small-sample, single-center, single-ethnicity studies with a lack of external validation are clearly insufficient to deeply explore sex differences in asthma at the metabolic level.

Multi-omics clearly needs to combine information from multiple omics layers to gain a deeper understanding of the asthma disease process. However, most studies so far have only attempted to pair information from two omics layers (e.g., genomics and transcriptomics, proteomics and metabolomics) for linkage, with few attempts to integrate more layers. Integrating multi-layer omics information undoubtedly faces challenges in linking representations and handling large amounts of data and noise. Introducing machine learning and deep learning algorithms can greatly address these issues. Such attempts have indeed achieved some success (Li et al., 2018; Poirion et al., 2021; Chaudhary et al., 2018), but these algorithm architectures do not imply that one method is always superior to others. Different methods can study data from different angles, and different types of data require different methods. This places higher demands on the quality of omics data and the standardization of experimental methods. Moreover, integrating omics data with clinical and demographic data introduces additional complexity. The integration of multi-modal data is essential to obtain a more comprehensive, detailed, and precise patient perspective, which is crucial for achieving true precision medicine. As a complex disease, asthma’s sex differences are also influenced by sex hormones, with prevalence rates varying across age groups and physiological cycles. Given the dynamic nature of asthma and its symptoms, cross-sectional studies are often insufficient to capture the relevant information. Therefore, more longitudinal studies are needed to better understand the prediction, prevention, and personalized treatment of asthma.
